# Academic and clinical perspectives of metagenome sequencing as a diagnostic tool for infectious disease: an interpretive phenomenological study

**DOI:** 10.1186/s12879-025-10820-x

**Published:** 2025-03-31

**Authors:** Hannah Trivett, Alistair C. Darby, Oyinlola Oyebode

**Affiliations:** 1 Health Protection Research Unit in Gastrointestinal Infections, University of Liverpool, Liverpool, UK; 2 Institute of Infection, Veterinary and Ecological Sciences, University of Liverpool, Bioscience Building, Liverpool, UK; 3 Institute of Microbiology and Infection, College of Medical and Dental Sciences, University of Birmingham, Birmingham, UK; 4 Wolfson Institute of Population Health, Queen Mary University of London, Mile End Road, London, UK

**Keywords:** Clinical metagenomics, Interpretive phenomenological analysis, Healthcare professionals, Infectious disease diagnostics, Microbiology

## Abstract

**Background:**

Effective infectious disease diagnostics (IDD) are vital for informing clinical decision-making regarding the treatment and patient management of disease and infections. In England, conventional clinical methods rely upon culture-dependent techniques, and there has been little shift in the acceptance and integration of culture-independent sequencing methods into routine clinical IDD. This study explored stakeholders’ experiences within IDD, including those working in clinical settings and those conducting research at the forefront of microbial genomics. From the participants’ experiences, the study aimed to identify barriers and facilitators driving the development and implementation of metagenome sequencing as a routine diagnostic.

**Methods:**

Virtual semi-structured interviews were conducted with purposively selected individuals involved in IDD. The interviews explored the experiences of implementing metagenome sequencing as a diagnostic tool and decisions about which diagnostics are used for identifying bacteria-causing infections. Thematic analysis was used to analyse the data, and an Interpretive Phenomenological approach was used throughout.

**Results:**

Ten individuals were interviewed between July 2021 and October 2021, including clinical scientists, consultants, and professors in academia. Their experience ranged from limited knowledge of metagenome sequencing to an expert understanding of the phenomenon. The thoughts and perspectives of participants of the study could be grouped into five themes: Availability of diagnostics for infectious diseases; Clinical laboratory infrastructure; Ethical Data Sharing: Enhancing metagenomics through Open Access; Case study in action: COVID-19; and The importance of communication to improve developments of new diagnostics. Participants recognised the need for new diagnostics to be implemented to overcome the limitations of current diagnostic approaches but highlighted the barriers to integrating new diagnostics into clinical settings, such as the impact on clinical decision-making, accreditation, and cost. Further, participants felt that lessons could be learnt from using metagenomics in COVID-19 and how other diagnostic platforms have been integrated into clinical settings over the last 20 years.

**Conclusions:**

The study provided insights into stakeholders’ perspectives and opinions to address the knowledge gap in current literature and identified barriers and facilitators which drive the implementation of metagenome sequencing as a routine IDD in clinical settings. Knowledge of new and upcoming genomic diagnostic testing is not equally distributed throughout the UK, impacting the understanding and drive to integrate metagenome sequencing into routine clinical diagnostics. Improvements in access to new diagnostics could improve patient treatment and management and positively impact population health.

**Supplementary Information:**

The online version contains supplementary material available at 10.1186/s12879-025-10820-x.

## Introduction

The term metagenomics was first published in 1998, referring to directly sequencing a collection of genes from a sample and analysing similarly to a single genome [1]. Shotgun metagenomics uses a hypothesis-free, unbiased approach to study the structure and function of the entirety of an environment’s nucleotide sequences, including parasites, fungi, bacteria and viruses. The technological advancements in sequencing have made metagenomic next-generation sequencing (mNGS) an attractive choice for clinical personnel who want to apply metagenomic methods to infectious disease diagnostics (IDD).

Current clinical diagnostic methods for bacterial pathogens are often culture-dependent, requiring the growth and isolation of organisms using methods such as selective culture media growth, biochemical testing, and Matrix-assisted laser desorption-ionization time of flight mass spectrometry (MALDI-TOF), in addition to molecular techniques like Polymerase Chain Reaction (PCR) [2]. However, they are limited by the need for a priori knowledge of the potential pathogens present, and MALDI-TOF is limited by the availability of organisms represented in curated databases to determine the pathogen present within the sample [3]. In addition, in some cases, such as those involving slow-growing organisms, it can take as much as two weeks to cultivate a sample, inhibiting the rapid diagnosis and treatment of some infectious diseases.

mNGS can overcome the current limitations of culture-based ‘gold standard’ methods used today in clinical laboratories. Unlike the targeted diagnostic methods identified above, mNGS is a hypothesis-free approach which enables the simultaneous identification of microbial taxa from a single sample without prior pathogen assumptions. This unbiased method of pathogen detection has demonstrated its utility in challenging clinical scenarios, including culture-negative infections, immunocompromised patients, and coinfections in critically ill patients, where traditional diagnostics often yield inconclusive results [4–6]. These studies have demonstrated the utility of mNGS in identifying the causative agents of infections without the need for confirmatory whole genome sequencing or culture-dependent streaking and biochemical tests. The typical mNGS workflow consists of several key steps: sample collection, nucleic acid extraction, library preparation, sequencing and bioinformatic analysis. Additional steps can be added, such as host-depletion to remove host (human) DNA to increase microbial sequence abundance [7]. After sequencing reads are generated, the reads are subjected to bioinformatic analysis, often in the form of pipelines such as EPI2ME [8], DRAGEN [9], or BugSeq [10]. These tools facilitate classifying microbial taxa, functional gene analysis and identifying virulence and antimicrobial resistance genes. Technologies like Oxford Nanopore offer “real-time” profiling of bacterial genomes for rapid and accurate pathogen detection, enabling rapid access to time-critical results. A culture-independent method of surveying the sample site of infection enables clinicians to capture novel pathogens, which may go undetected when using diagnostics such as PCR, which are limited in detection capabilities based on their primer scheme (11). The cumulation of millions of bacterial sequences provides scope for mNGS to act as a toolkit identifying pathogens responsible for infections and capture functional gene profiling and virulence gene identification from a single extracted sample. In addition, metagenomics has been documented to have the potential to predict antimicrobial sensitivity by identifying antimicrobial resistance genes in real-time, which allows for faster determination of antimicrobial sensitivity. Delays in culture-based diagnostics, such as phenotypic sensitivity testing, often lead to the prescription of broad-spectrum antibiotics speculating the appropriate treatments for bacterial infections. Metagenomics potentiates the reduction in the dependency on broad-spectrum antibiotics. It enables clinicians to prescribe targeted therapies earlier in the treatment process by identifying pathogens and their associated resistance genes in a clinically relevant timeframe of less than 24 h [12].

Yet, whilst mNGS offers the potential to revolutionise IDD with a suite of clinical and surveillance applications, there has been a shortfall in early adoption to bring this diagnostic into clinical use, which may be due to several limitations such as lack of expertise, cost and lack of standardised approaches [11, 13]. Rather than replacing existing diagnostic workflows, mNGS has the potential to complement current diagnostic methods by serving as an adjunct tool. This approach is particularly valuable in cases where conventional methods are inconclusive or impractical, such as culture-negative infections, polymicrobial infections, or when patients have received prior antibiotic treatment that may limit pathogen recovery.

The utility of metagenomics is often explored with case studies, pilot data, and systematic reviews. In studies using pilot data, the findings are not truly representative of the experiences within clinical settings. Using quantitative research sheds light on metagenomic sequencing utility; however, there is a lack of understanding of its effectiveness, strengths and weaknesses within clinical settings. Whilst it is important to provide case studies as evidence for metagenomic sequencing usefulness in a quantitative manner, qualitative methodologies can provide a more detailed context of the implementation of IDD. Why clinicians are not utilising the latest mNGS methods is under-researched, with a lack of qualitative research to understand the current diagnostic landscape and to evaluate why the landscape has remained unchanged for many years. Current literature is saturated with publications on clinical metagenomics, covering topics such as its utility and its limitations when applied to the current diagnostic landscape [14–16]; however, there is a gap in the literature which contextualises the recent clinical advancements in IDD and the barriers that hinder the implementation of new diagnostic frameworks. Perspectives and opinions of clinical diagnostic stakeholders influence the uptake of new diagnostic technologies [17]. Qualitative research can complement quantitative research methods by bridging the gap between numerical data from research and public health practice, providing the context necessary for a comprehensive understanding of a phenomenon [18]. A phenomenological approach enables researchers to learn from the lived experiences of others. An ideographic line of enquiry empowers the researcher to understand the various factors that influence healthcare professionals’ perspectives and experiences of clinical diagnostics, uncovering meanings through interpreting events and experiences. Therefore, collecting qualitative evidence and taking a phenomenological approach to understanding the utility of mNGS in clinical settings and the barriers to its implementation may allow innovations that will move diagnostics into the next-generation sequencing era.

This study aimed to explore the opinions and views of individuals with IDD on the utility of mNGS and the barriers and facilitators impacting the future development and integration of genomic diagnostic methods for routine clinical use.

## Methodology

The qualitative enquiry undertook an interpretive phenomenological approach (IPA), documenting the lived experiences of stakeholders participating in the study. IPA is an ideographic approach that emphasises the personal sense-making of the experiences. IPA is a helpful approach many healthcare researchers adopt to allow others to learn from individuals’ experiences whilst also acknowledging the subjectivity of interpretation, that the researcher’s experiences may impact interpretation [19, 20]. The reporting of this study was facilitated by using the consolidated criteria for reporting qualitative research (COREQ) (Supplementary file 1) [21].

The study gained ethical approval from the Institute of Life Course and Medical Sciences Research Ethics Committee at the University of Liverpool (Ethical review reference 9855).

### Author reflection

The research team was made up of three individuals, including two women and one man. At the time of the study, HT was trained to BSc in Microbiology and was undertaking a PhD in Microbial genomics, supervised by AD and OO, who hold PhDs and are professors in Public Health (OO) and Genomics (AD). HT and OO had training, and OO had previous experience in conducting qualitative data collection. The research team described the reasons for doing the research within participant recruitment emails as being to understand the current landscape of clinical diagnostics for infectious diseases and the added value of clinical metagenomics in clinical laboratories, to support the integration and development of clinical metagenomic pipelines fit for routine clinical use.

### Participant recruitment

**Fig. 1:**
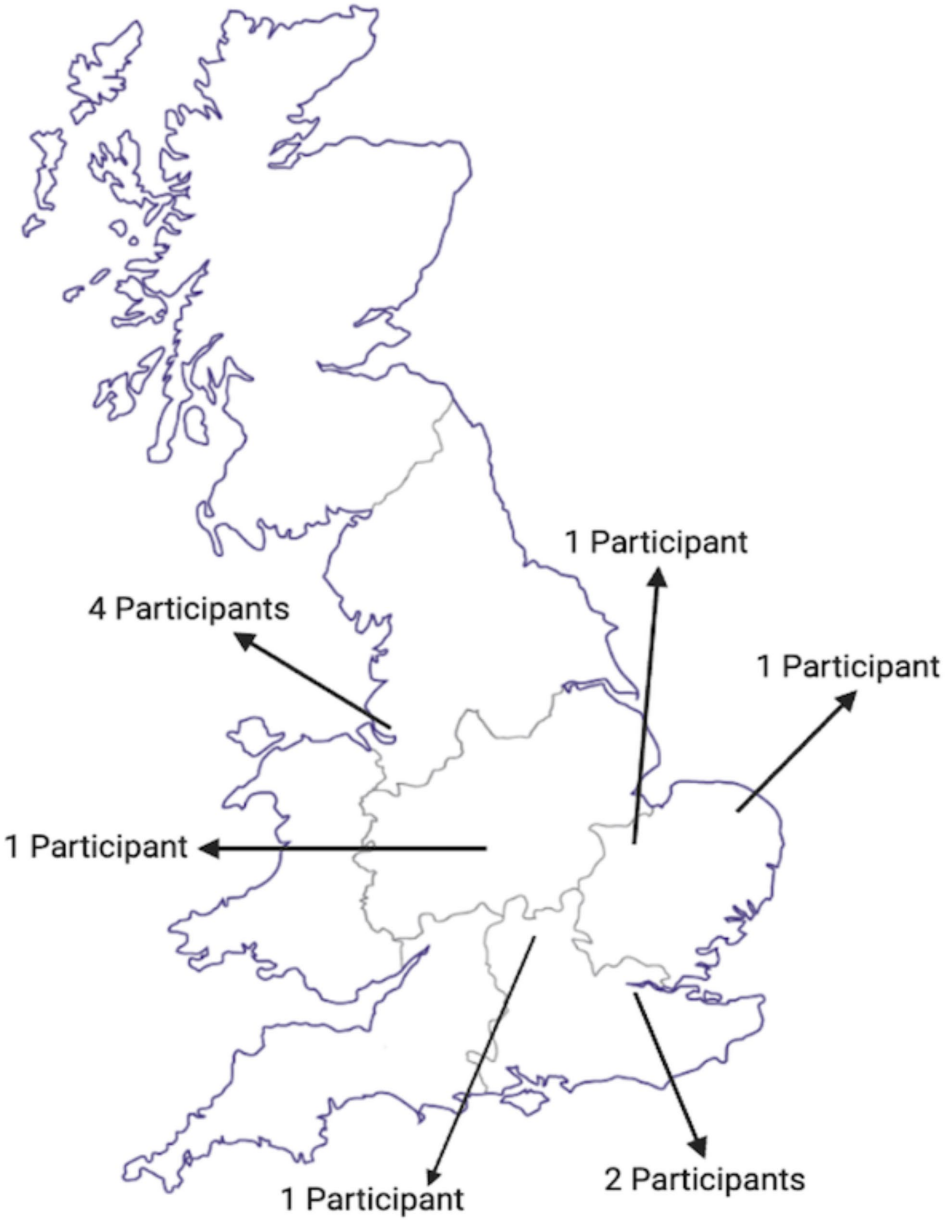
Map of participants’ UK locations. 2 participants are based in London, 1 Participant is based in the Southeast, and 2 participants are in the East of England. One participant was in the West Midlands, and four were in the Northwest

Participants were purposively recruited across England to ensure information-rich clinical perspectives, with various viewpoints and experiences. The participants were recruited through emails sent out to clinicians and academics within the researchers’ networks. Efforts were made to recruit participants from different professional roles within academia and clinical settings, across geographical locations in England, and at different career stages to encompass a breadth of viewpoints. Additionally, participants were selected to reflect a range of service settings, including hospital-based laboratories and academic institutions. This approach aimed to ensure that the data captured both strategic and operational perspectives on the phenomenon under study. Participants had differing levels of epistemology of the phenomenon to ensure various experiences were covered, as well as spanning geographical location, department, length of service, and profession (Table 1; Fig. 1). Level of knowledge of metagenome sequencing applied IDD was self-identified by each participant. Self-identification of expertise was based on participants’ own assessment of their knowledge and experience of metagenome sequencing applied to IDD. To provide further context, participant demographics, job roles, and self-reported expertise are presented in Table 1. IPA informed the participant recruitment number as IPA requires a small sample size of around 5–10 participants, allowing for an in-depth analysis of everyone’s experiences [22]. The target number of participants for the study was 10, there was no specific goal for the number of individuals to be interviewed per profession, as COVID-19 impacted availability of participants to interview.

**Table 1:** Participant demographics and self-identified knowledge

	Gender	Profession	Self-identified knowledge of metagenome sequencing applied to Infectious Disease Diagnostics
**P1**	Female	Consultant clinical scientist and academic researcher	Expert knowledge
**P2**	Female	Academic researcher and physician	Expert knowledge
**P3**	Male	Infectious disease consultant	Some knowledge
**P4**	Male	Consultant and academic researcher	Good Knowledge
**P5**	Male	Academic researcher and physician	Expert knowledge
**P6**	Male	Consultant clinical scientist	Good Knowledge
**P7**	Female	Infectious disease consultant	Limited Knowledge
**P8**	Male	Clinical director	Limited knowledge
**P9**	Female	Academia	Expert knowledge
**P10**	Male	Academia	Expert knowledge

### Data collection

Semi-structured interviews took place via Microsoft Teams. HT conducted interviews between July 2021 and November 2021. The semi-structured interview guide was created, informed by a literature review, to allow the interviewer to guide the line of questioning—the open-ended questions provided flexibility in exploring topics that may emerge in interviews. IPA informed the interview schedule to ensure open-ended questions allowed for in-depth explorations of participant experiences in the current diagnostic landscape and their opinions of the integration of metagenomic methods into clinical space as a diagnostic. The interview guide was checked by two authors (OO and AD) and piloted before beginning data collection with an individual independent of the study. A copy of the guide was sent to participants before their interviews (Supplementary file 2).

### Data analysis

Interviews were video and audio-recorded, and the data were transcribed verbatim in Microsoft Word and analysed using an IPA by HT [2]. All data were anonymised, and inductive thematic analysis was conducted [23]. Open coding was performed, identifying experiential statements for each participant, which were then grouped, looking for convergence and divergence between participant experiences; these groups formed the overarching themes of the qualitative enquiry, which were named using words closely linked to the data [24]. While we cannot claim data saturation across all professions due to the sample size per profession, consistent patterns and insights were observed across multiple interviews, suggesting thematic coherence within the data. A draft of the findings was made available to participants to allow them to check over the study to ensure the information accurately represented their views. All participants were happy with the draft of the findings.

## Results

**Fig. 2:**
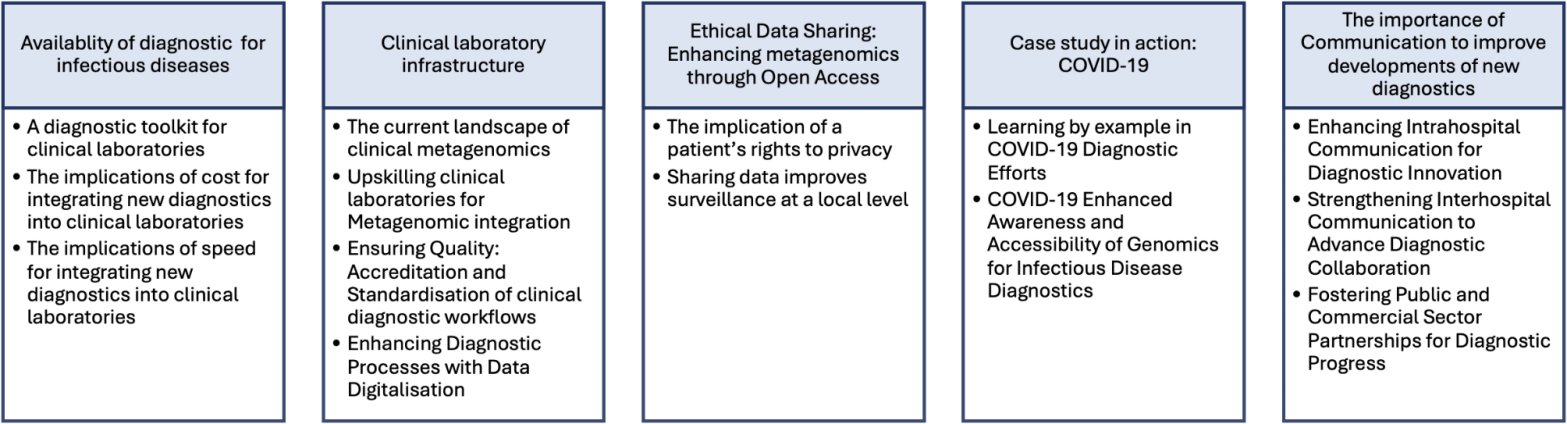
Themes and Subthemes

**Fig. 3:**
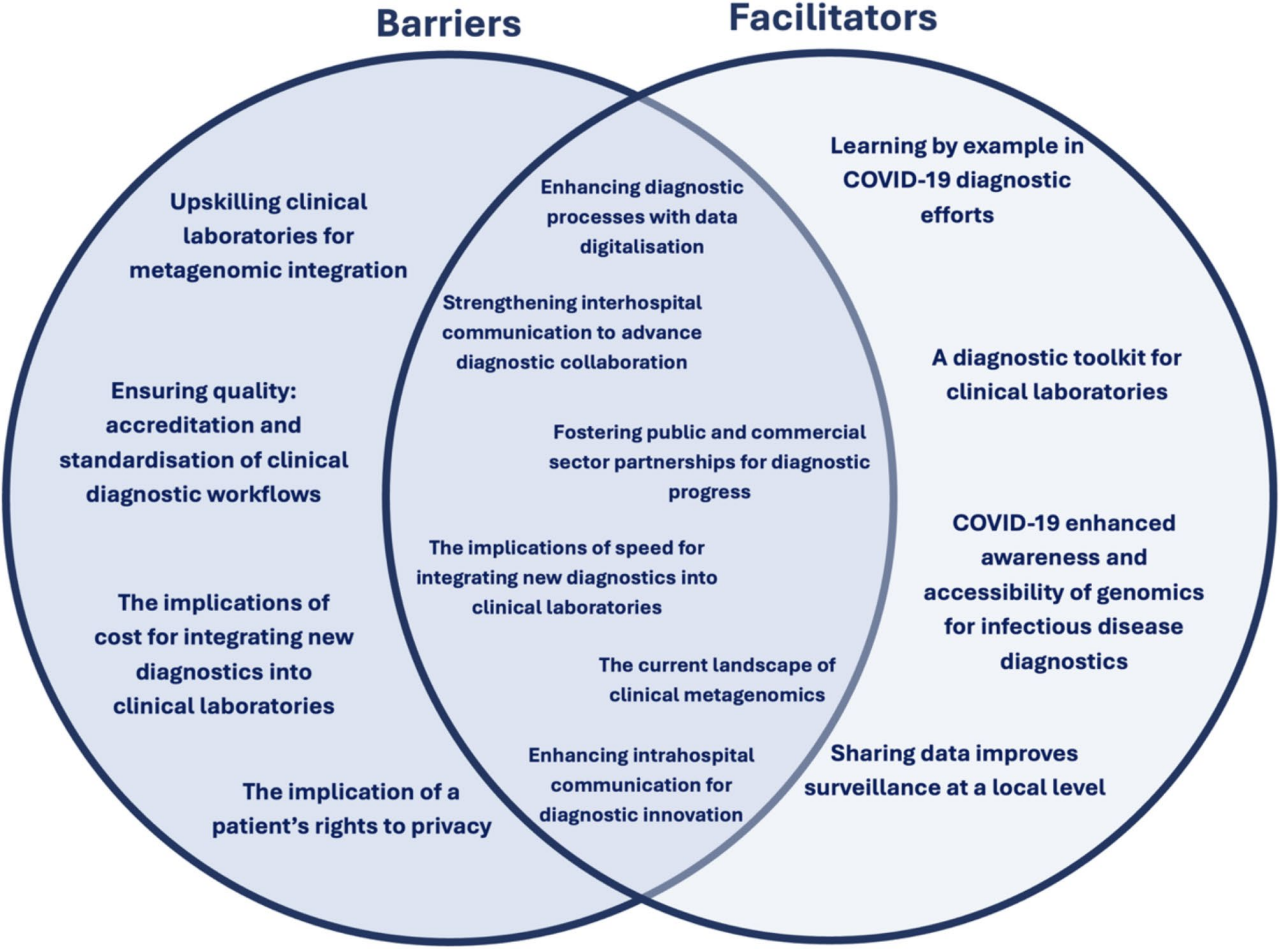
Schematic partitioning of subthemes as barriers and/or facilitators to implementing metagenomics as a clinical diagnostic in infectious disease laboratories

Ten participants participated in interviews lasting between 30 and 60 min. Participant characteristics are shown in Table 1. Five themes, with associated sub-themes, were identified from the interviews with all participants (Fig. 2). Reflecting on the insights provided by participants, sub-themes were categorised as barriers and/or facilitators that impact the implementation of metagenomics as a routine clinical diagnostic for infectious diseases (Fig. 3).

### Availability of diagnostics for infectious diseases

The availability of diagnostics for infectious diseases was highlighted as an important concept that encompasses the variety of methods used to identify pathogens causing infection, as it ensures timely and accurate pathogen identification, imperative for guiding patient treatment and infection control measures. Participants offered a range of examples currently available. However, the availability of tools was subjective to each hospital. Some hospitals have a whole suite of diagnostics, including genomics, serology, and MALDI-TOF, while other hospitals are limited to culture swabbing to screen for infections and limited PCR.

#### A diagnostic toolkit for clinical laboratories

Throughout the interviews, participants reinforced the benefit of introducing mNGS into clinical laboratories, providing another tool to the armoury and increasing the power of choice to suit clinical needs. However, participants did highlight that mNGS diagnostics would not be a one-size-fits-all approach. Instead, there would be a suitable place and right time to use within the spectrum of tools currently available. Clinical scientists and consultants would have to learn when the appropriate time to use metagenomics to diagnose would be.

*P4 “There are new molecular tests*, *and actually*, *there is a steady stream of the introduction of new things to do in response to clinical need. However*, *there is a set of technologies that are finding it harder to get into the clinical place for infectious diseases*, *of which genome sequencing is one.”*

*P1 “I don’t think it’s a panacea*, *I think it’s very much part of our toolkit*, *and there will be things that are appropriate to use it for*, *and there will be things that are less appropriate for it to be useful. We have to see it as not one or the other but as a spectrum of clinical choices in terms of diagnostics where*, *just like everything else*, *I choose the right test for the right question.”*

#### The implications of cost for integrating new diagnostics into clinical laboratories

There was a broad consensus that cost significantly contributed to the choice of diagnostics on offer, which in some cases impacted the development and integration of metagenomics for routine clinical use. Whilst the affordability of genomic machinery and library preparations has reduced considerably since sequencing first came on the horizon, many commercial providers of diagnostic platforms are still directed towards the research sector, where funding is far more accessible through research grants; thus, distribution is geared towards those who can pay. Several participants stressed that their ability to introduce sequencing into the clinical diagnostic frameworks was due to the procurement of research grants rather than funding available directly from the Trusts or government.

*P2 “All of the sequencing we did was based on a research budget*, *so we need*, *we need substantial funding from the NHS in order to roll this out*, *that’s the first thing.”*

*P1 “If I was working in a normal setting and I wasn’t a researcher*, *then I wouldn’t have access to the training*, *knowledge*, *understanding and money it takes to bring these things in to get the data that you need to be able to transition it. So*, *most of our work happens because of the fact that I have research funding.”*

Disparities were seen between smaller district hospitals and those in larger cities with greater access to funding. Participants from smaller hospitals expressed frustration over limited funding, in contrast to city-based hospitals with greater financial support, highlighting the inconsistency in the availability of diagnostics across the UK. Collectively, participants deployed in clinical settings commented on the need for equitable distribution of diagnostics to provide the same level of clinical care for all patients.

*P1 “If you’re going to make a change across the board*, *we can’t be disadvantaging somebody in South Devon just because of where they live. We need to be doing this in an equitable fashion where those that need it have access.”*

Throughout the interviews, the distribution of money was identified as a critical limiting factor to implementing new diagnostic frameworks, requiring a business case model to reflect the cost-effectiveness and patient benefit of introducing metagenomics. Participants within clinical settings acknowledged the need for better collaboration between those on the laboratory frontline and health economists to bring about a strengthened argument to advocate for the successes of sequencing and the direct impact introducing new diagnostics would have on relieving the patient pathways.

*P8 “It’s been very painful trying to bring those methods on board*, *and I think it’s been very painful trying to demonstrate the overall cost-effectiveness. All people look at what the acquisition costs are in pathology*, *and we don’t have enough money*, *and it’s just taking that whole health economy view*, *which we are*, *I think.”*

#### The implications of speed for integrating new diagnostics into clinical laboratories

While cost was a significant barrier to implementing metagenome-based diagnostics, the speed of obtaining actionable clinical results from metagenomic methods was also a key factor in why many hospitals do not offer them in their laboratories. If new technologies can bring about enough change, funders are willing to pay.

*P1 “If it gives you enough of a management change*, *you can usually find a payoff; what you can’t do is fix time … If I’m not getting it in a clinically actionable time frame*, *then it is of interest*, *it is not of use.”*

*P4 “There needs to be a distributed model for the advancement of infectious diseases diagnostic capability*, *where the testing is done as close to the front line as possible… I think you need to have a more local approach with infectious diseases and testing because things need to be faster*, *and people need to understand what the clinical implications are.”*

The importance of speed was reiterated when addressing technology’s overall impact on guiding treatment and patient management. Culture-independent methods of diagnosing infectious diseases must reduce the turnaround time between a patient presenting with symptoms and delivering the correct therapy in response to identifying the causative agent. Participants identified that whilst the processing of samples may be relatively short, the time the sample is on the machine may be too long to provide treatment in a clinically actionable time frame, limiting the uptake in implementing metagenomics as a diagnostic.

*P4 “If your result is going to take more than five days to come back*, *it’s probably irrelevant. I mean*, *it’s academically interesting*, *but it’s not going to guide therapy.”*

### Clinical laboratory infrastructure

Infrastructure embodies the facilities and systems required to establish metagenomic sequencing in clinical settings. The participants’ perspectives on infrastructure included physical laboratory space, digital space (such as computational setup and storage), and the workforce and training; collectively, all parts of infrastructure must be addressed for the successful integration of metagenomics.

#### The current landscape of clinical metagenomics

Participants reflected on various barriers they experienced, stalling the integration of metagenomic diagnostic frameworks. Although participants could see the potential benefits of introducing point-of-care metagenomics, they commented that they had seen a slow uptake with new technologies, lagging behind use in research- many believed they did not have the infrastructure to facilitate implementation.

*P6 “It’s still so specialised*, *and should it be? Or should we be able to be offering this as a wider method? And I think*, *I really think we should be; we just don’t seem to be able to get across that hurdle.”*

Current UK-centralised models of whole genome sequencing and metagenomic analysis delay response time for directed therapies due to the extended turnaround time of shipment, sequencing, and relaying information to the clinical microbiology team. The bottleneck impacts clinicians’ decision-making and could have potential implications of adding to the antimicrobial resistance crisis by administering antibiotics that are not purposefully prescribed for specific bacterial infections. Current responses are primarily to administer empirical treatments before the pathogen is identified. This can prolong the patients’ treatment, widening the gap between admission and treatment with specific narrow-spectrum antibiotics better suited for the infection.

*P7 “I think it’s really important because now we send [a patient sample or isolate] for typing and we get a result*, *I don’t know*, *three weeks later*, *by which time you know you’ve already acted on basis of your clinical suspicion.”*

Decentralising laboratories and providing local infrastructure can overcome many of the obstacles documented by the individuals in the study. One participant highlighted a successful model in another country where mNGS was integrated into local hospital laboratories, significantly improving turnaround times. The infrastructure for mNGS offered solutions to barriers seen in the UK, such as turnaround time when metagenomics and genome sequencing were local to the patients.

*P9 “In Germany*, *[there are] small regional centres as well as the Robert Koch Institute in Berlin*, *so they can respond much more quickly in terms of turn-around time because they have all those local labs.”*

#### Upskilling clinical laboratories for metagenomic integration

Currently, Biomedical and Clinical Scientists have few opportunities to access certified training in bioinformatics, a vital skill for interpreting genomic data to impact clinical decisions for the treatment of patients. Participants indicated that re-training and educating current laboratory personnel is essential for the uptake of new diagnostic frameworks, such as metagenomic sequencing, which is unavailable or has limited availability to clinical staff.

*P9 “I think it would be invaluable for the diagnostics team to have at least some level of [bioinformatic] knowledge because I think that it empowers people also to have the confidence to interpret any data that comes through.”*

Participants based within clinical laboratories expressed that the current array of skills within clinical laboratories lacks the expertise required to establish a full mNGS workflow. Currently, many laboratories rely on local collaborators to interpret bioinformatic data, which has implications for clinicians receiving data within a clinically actionable timeframe. Introducing new team members, such as bioinformaticians, would alleviate the pressures of outsourcing and thus overcome another barrier to implementing metagenomics.

*P3 “One of the things that we are struggling with is that we’re relying on bioinformatic support from elsewhere and not having that on-site.”*

However, several individuals acknowledged that workforce staffing levels could be better and that introducing new metagenomic diagnostics frameworks may be beyond the current scope of these staff numbers.

*P6 “If we are introducing an entirely new workflow*, *it has a staffing implication and generally*, *labs are poorly staffed at the moment.”*

#### Accreditation and standardisation of clinical diagnostic workflows

Participants in microbial laboratories shared the constraints they experienced when bringing new diagnostic techniques to test, validate, and roll out. Many reagents are labelled as ‘research use only’, which restricts clinical usage as they do not qualify for medical application. This limits the availability of reagents that are permitted for medical purposes.

*P1 “Accreditation is a nightmare*, *so all of our tests have to be accredited*, *so they have to be ISO accredited by a group called UKAS who come in and assess us. All of the reagents that are used for whole genome sequencing and metagenomics say they’re for research use only*, *and therefore*, *to get accreditation with a whole bunch of research reagents is incredibly difficult.”*

In addition, providing evidence for the standardisation of diagnostics processes could be improved. One individual highlighted that many clinical samples are small in quantity, meaning processes cannot be standardised with the same sample; data would be varied with no standard control to validate against. The metagenomic pipeline for diagnostics is far-reaching, applying to various sample types and microorganisms; however, participants identified this as challenging. The variety of outcomes from analyses could complicate the standardisation processes as each sample would, in principle, need its validation.

*P10 “I think you could validate metagenomics diagnostic service*, *but the outcome would probably not be a diagnosis. The outcome would probably be a data set*, *you know*, *because it would be hard to put in a random sample*, *and you come out with an answer because we just don’t have enough.”*

#### Enhancing diagnostic processes with data digitalisation

During the interview process, there were frequent discussions of inadequate internal laboratory systems used for data storage for storing large data sets produced by genomic machinery. Many organisations have begun to digitalise their data storage and management system infrastructure to access patient data, streamlining data accessibility. With extensive data footprints in genomics, IDD departments should engage with other departments to learn from others’ experiences in digitalisation to help integrate and develop genomics in healthcare.

*P8 “This sort of data won’t fit into most laboratory systems*, *I would suggest. So*, *there’s a lot of work ongoing at the moment about digitising cellular pathology*, *and you might want to piggyback onto that to find ways of getting this data stored.”*

The infrastructure required for the technology spans the whole pipeline from physical laboratory space to data storage to technology for clinical interpretation. Participants questioned their ability to understand and clinically interpret the data. Participants flagged the need for infrastructure that is easy for individuals to get to grips with and that can provide the correct information that is clinically usable, not just laboratory infrastructure and data storage.

*P7 “I think all technology has to be right*, *not just the assay*, *but the reporting side of things. But we need kind of more insight as to how to report them in a clinically useful way.”*

### Ethical data sharing: enhancing metagenomics impact through open access

Genomic data is often open access and widely shared within the research community. Having data openly available and easy to access accelerates research to find new ways of testing new hypotheses and developing new analysis methods. Participants recognised that challenges are met by the ethical considerations of a patient’s right to privacy versus sharing healthcare data to learn and develop genomic expertise for wider public health benefit.

#### The implication of a patient’s rights to privacy

Open accessibility and the ability to share data are core principles for developing and understanding infectious diseases. However, patient information from clinical records is confidential, and restraints are in place to maintain patient privacy of personal information. Here, we see a conflict between the core principle of healthcare systems respecting a patient’s right to privacy and the ability to expand our public data repositories to keep up with evolving infectious diseases and knowledge of diseases.

*P10 “Genomics comes from an open data kind of culture*, *but clinical diagnostics most certainly doesn’t for obvious reasons*, *and so does public health epidemiology. So*, *there’s a culture clash there between whether genomes are public goods*, *which should be deposited in the public database as soon as you get them*, *versus people’s rights to privacy for their diagnosis*, *and this is the general culture of public health which is more closed which may need changing a little bit.”*

#### Sharing data improves surveillance at a local level

A comprehensive application of data sharing impacts individual patients for diagnostic purposes and disseminates knowledge and understanding of the disease and epidemiology, guiding therapeutic development. Participants highlighted the positive impact of local data generation fed into a centralised data repository. This data can guide disease surveillance and help us better understand infectious diseases and their evolution, similar to what was seen from the COVID Genomic UK Consortium (COG-UK) and the developments in the knowledge of COVID-19.

*P2 “Data generated locally can be collected for national surveillance*, *and that’s an amazing opportunity…. If you had a centralised database*, *you could get some amazing insights into what’s happening across the country.”*

### Case study in action: COVID-19

COVID-19 was one of the largest modern-day international outbreaks documented, and the use of genomics to identify and survey the virus is a prime example of the utility of metagenomics within public health. Collectively, participants commended the use of metagenomics in this outbreak, offering several benefits they saw from its implementation worldwide.

#### Learning by example in COVID-19 diagnostic efforts

Participants offered an insight into their experience and opinions of the capabilities of utilising genomics. They believed the COVID-19 model provided proof of concept for further integration as a diagnostic in a clinical setting. Such data-sharing frameworks can strengthen infectious disease surveillance, enhance public health responses, and inform targeted interventions.

*P2 “SARS-CoV-2 is an exemplary example of where sequencing can bring impact to public health and individuals*, *so it’s gone beyond proof. It is being used every day and based on that*, *and I don’t think there’s any going back to thinking that sequencing is some sort of luxury*, *and so now we need to work out where it’s going to be used when it’s going to be used.”*

Participants highlighted that in recent years, there had been an uptake in routine whole genome sequencing for several pathogens within the centralised model of the genome sequencing service provision provided in the UK, including Tuberculosis and Salmonella. Most recently, metagenomics was used to identify COVID-19, providing an excellent example of the positive impact metagenome sequencing can have on public health.

*P10 “The COVID experience*, *I think*, *pushed the argument forward for routine use of sequencing*, *as nothing else has done. There are routine uses of genomics now for TB*, *Gram-negative foodborne pathogens like salmonella*, *HIV*, *hepatitis maybe*, *but those are the exceptions rather than the rule of thumb.”*

#### COVID-19 enhanced awareness and accessibility of genomics for infectious disease diagnostics

The influx in demand for sequencing technology and reagents from COVID-19 sequencing meant that an increase in manufacturing capacity was established. Increased production of reagents and sequencing platforms has improved the accessibility of consumables to medical laboratories, increasing the likelihood of integrating new diagnostic frameworks into IDD departments.

*P1"The big advantage of all the stuff that’s going on for me in the whole genome sequencing world is that it is driving manufacturers to make stuff that is useful for me. So even though I’m not getting the data*, *the benefit will be that I now have like the next evolution of Nanopore which means that I can run one or two samples instead.”*

Participants expressed positivity towards what COVID-19 sequencing platforms had done for the visibility of genomic sequencing, pushing forward the case for implementing metagenome sequencing as a routine clinical diagnostic.

*P5 “The pandemic and the experiences with sequencing SARS-CoV-2 bought it into very sharp focus*, *and that it has motivated not only governments and health agencies but indeed has started to resonate with the public.”*

### The importance of communication in improving the development of new diagnostics

Participants collectively acknowledged that communication was a driver for implementing mNGS diagnostic frameworks in clinical laboratories. They identified three key relationships facilitating this: communication channels within hospitals, between hospitals, and between hospitals and other organisations, such as academia and commercial businesses.

#### Enhancing intrahospital communication for diagnostic innovation

Intrahospital communication is defined as the sharing of information within one institution. UK hospital organisation sees departments running independently of one another, with pots of money funding departments in silos and not equal between different disciplines. With departments working autonomously, there needs to be more communication between groups, which can make healthcare disjointed.

*P9 “hospitals are really bad at speaking between disciplines in different clinical care.”*

One participant compared their experiences of working in UK and German hospitals. Communication channels within hospital departments were more established, with crosstalk between departments, utilising genomic machinery that other departments already have in play. Collaborating within organisations provided efficiency in integrating new technologies, rapidly adopting new diagnostic workflows, and positively impacting patient management and treatment with the latest technology.

*P9 “I walked around the big paediatric hospital here in [German city]*, *so this was with the infectious disease paediatrician. He took me to the guys that all do the rare diseases*, *and he goes*, *‘we just use all of their Novaseq to do our sequencing. Why would we buy it when they’ve got it down the corridor?’ and I’m like*, *100%*, *but I was like a bit like*, *you know*, *mind blown because I was like*, *this doesn’t necessarily happen in other situations.”*

#### Strengthening interhospital communication to advance diagnostic collaboration

Interhospital communication is the sharing of information between multiple sites of clinical organisations. Participants highlighted that early adoption of metagenome sequencing will likely be in larger hospitals. The knowledge and experience of setting up and establishing frameworks should be disseminated to other sites to streamline the integration of metagenomics for other organisations.

*P4 “Other places*, *each one will have their journey*, *and so it just needs to be focused on. We need to learn from a few sites that have started to do it and listen to them like the conversation we’re having now and share experiences.”*

However, other participants believed not all hospitals easily communicate their experiences to other microbiology diagnostic teams to help others transition to new mNGS clinical diagnostics.

*P1 “I just get really concerned that all of these things happen in pockets and by people do stuff in pockets without a strategic view*, *without them feeding back to the right places. Then actually*, *it’s just all of us repeating work.”*

A few participants focused on their positive collaboration experience during COVID-19, where effective communication between local hospitals and COVID-19 sequencing hubs improved collaborative efforts. This streamlines responses, the use of genome sequencing, and learning from the experiences of others to enhance the integration of these technologies.

*P6 “I think it’s been really helpful*, *particularly in our network*, *there was the weekly meetings have been very useful for disseminating information about what we need because there was so much change happening; being able to deal with that as a group is better than everyone just doing their own thing and essentially duplicating work to deal with problems.”*

#### Fostering public and commercial sector partnerships for diagnostic progress

Involving all participants in implementing new diagnostics for clinical use is vital for innovation. Participants collectively commented on better communication between all parties engaged in clinical diagnostics.

*P9 “Definitely a disconnect between the scientists that are maybe trying to drive this forward versus the people that are actually in the clinic. I think improving that communication between them is really important when we do this.”*

Participants within clinical settings highlighted that metagenomics is not the first technology system integrated into clinical settings, calling on their experience of MALDI-TOF integration into clinical laboratories. Integration and the development of new technologies require clear communication channels to produce clinically relevant platforms and solve current problems microbial laboratories see today in diagnostics. Improving communication between all parties, academia, industry, and healthcare, would increase productivity in developing clinically relevant products that are fit for purpose.

*P8 “Working with manufacturers is important*, *isn’t it*, *because increasingly*, *we’re seeing the big manufacturers trying to set up whole systems for laboratories that integrate. So*, *there’s a blood culture machine*, *there’s a MALDI machine*, *and there’s automatic sensitivity testing*, *there’s maybe some PCR machines. So*, *it’s getting those manufacturers to develop the platforms to integrate metagenomics into the whole laboratory and that that would help move things forward as well.”*

*P4 “You need to align the funding bodies. We need to bring in all the stakeholders as equal partners…I think it needs an insightful*, *joined up*, *sort of mutually supporting and engaging partnership framework so that everyone can benefit from it.”*

## Discussion

The study aimed to gather the opinions and perspectives of IDD stakeholders, using their experiences to understand the current diagnostic landscape of infectious disease and the implications affecting the integration of metagenomics for routine clinical use. To the best of our knowledge, the findings outlined in this paper contribute to the first interpretative phenomenological account of experiences surrounding this topic. Beyond offering insights into perceived barriers and/or facilitators of integrating metagenomics into IDD, the findings contribute novel perspectives on the challenges stakeholders face in the clinical diagnostic landscape, such as organisational bottlenecks like availability of funds, upskilling workforces to be able to cope with the new demands of metagenomics, and technical considerations such as the ethical dilemma of genomic data being considered within the public or private domain, through the use of interpretive phenomenology. This paper also provides practical recommendations for overcoming these barriers, offering guidance for policy, workforce training, and the broader adoption of metagenomics in clinical settings.

The insights provided by participants presented a complexity of interrelated components that fell into categories of barriers and/or facilitators that impact the implementation of metagenomics as a routine clinical diagnostic (Fig. 3). Almost all participants shared similar perspectives on what they identified as barriers to implementing metagenomics in clinical settings. However, the extent of the impact of these barriers was subjective to the location of participants. These barriers directly correlate with the availability of funds, infrastructure, and relations with local research groups, universities, and commercial partners.

The study focused on England and covered six regions. Within the study, several barriers were region or participant-specific, such as access to appropriate laboratory infrastructure, training and access to funding. Often, these barriers can be associated with cost, which has been identified as a significant barrier in genomic and metagenomic integration into clinical settings. Whilst IPA cannot be used to generalise findings from participants sharing their experiences and perspectives, similarities in barriers and facilitators for implementation of metagenomic sequencing in clinical settings might be observed across the four devolved nations of the UK. As detailed in “United Kingdom: Health System Review 2022”, different grants are allocated to the healthcare administration teams in each of the four nations, with the responsibility for distributing these funds resting with commissioning groups, advisory boards, and healthcare providers [25]. These bodies determine the allocation of spending across various areas of healthcare, including infectious disease management and the rollout of genomics for diagnostics and surveillance. This can mean that financial barriers may be significant in countries where smaller grants are allocated to public health. In addition, public health agencies that advise healthcare policy differ across the nations, such as Public Health Wales, Public Health Scotland, UK Health Security Agency (UKHSA), and Public Health Agency in Northern Ireland. Whilst the four UK nations signed Shared Commitments for Genomic Healthcare, each of these agencies operates with distinct priorities, leading to variations in the implementation and focus of genomic strategies for IDD and surveillance across the UK [26]. For example, Scottish public health genomics is driven by the Genomics in Scotland: Building our Future [27], the Genomics Delivery Plan for Wales drives clinical genomics development in Welsh regions [28], and the Pathogen Genomic Strategy guides genomic implementation in England [29]. With inequitable public funding of genomics in healthcare inequitable, the implementation of genomics for routine diagnostics and surveillance remains uneven. It thus could influence the regional attitudes towards the importance of developing and integrating metagenomics into clinical settings for routine clinical use [30].

In addition to funding, the ability to communicate and collaborate with effective academic and commercial partners has a significant influence on the success of the development and integration of new technologies into healthcare settings, as reflected by participants throughout the study. This has been mirrored in recent publications, announcing pivotal partnerships between UK Biobank, Genomics England, National Health Service (NHS) England and Oxford Nanopore to devise and roll out a routine metagenomics programme for pathogen detection [24]. Oxford Nanopore Technologies has consistently been a leader in developing genomic technologies that are attractive to clinical settings due to agnostic pathogen detection and accessibility. In addition, the company regularly collaborates with clinical and academic groups to develop state-of-the-art diagnostics and surveillance technology. After several successful pilot studies led by Guy’s and St Thomas’ NHS Foundation Trust, an NHS Respiratory Metagenomics programme for rapid pathogen identification will be rolled out across hospitals in England [31, 32]. The regional focus of this Respiratory Metagenomics programme in England demonstrates the English-centric nature of many ‘UK-based’ studies for genomics-focused work. This planned rollout to a limited number of sites highlights the ongoing issue of geographical representation in implementing metagenomic technologies. Selective deployment raises concerns about equitable access and potential disparities in diagnostic capacity across different regions of the UK. These findings align with observations in this study, where participants identified regional disparities in infrastructure, funding, and the availability of partnerships as critical barriers to metagenomic implementation. For example, participants emphasised how the presence or absence of collaborations with leading academic and commercial groups significantly influenced the feasibility of deploying these technologies in their settings, as highlighted by P4 and P8 in theme 5. This suggests the barriers to implementation may be higher in the rest of the UK.

The qualitative data was supported by many of the current findings of literature reviews, which discuss similar barriers to metagenomics in clinical laboratories, including a lack of workforce training and expertise, workflow validation, and physical and digital space [11, 33]. However, this study goes beyond identifying perceived barriers from the literature. By actively engaging with professionals with direct experience with metagenomics and clinical diagnostics for infectious diseases, the study offers deeper insights into the practical challenges and opportunities in these areas.

There is a lack of a systematic approach to help level up IDD suites in clinical settings, which has impacted the uptake of routine mNGS at the local level. Many hospitals participating in pathogen sequencing rely heavily on external laboratories to carry out the workflow. With speed identified as a key barrier for implementing new diagnostics, transitioning away from centralised laboratories and moving sequencing to the source hospital could provide an opportunity to improve the speed of pathogen identification [34]. For cases where mNGS may not be local to the hospital, the practicalities of sending samples away for sequencing and waiting for results to be analysed outside of local hospital infrastructure can make mNGS inefficient in its current form as an external diagnostic. Removing time-consuming processes like preparing samples to be sent to external laboratories, mNGS could be faster than conventional culture-dependent bacterial identification. Offering a rapid turnaround from sample collection at the patient bedside to pathogen identification in less than 24 h would benefit public health through rapid diagnosis to inform patient treatment and public health management [35–37]. When developing new diagnostic methods, the whole workflow must be considered to ensure that methods are fit for purpose. Whilst a diagnostic workflow in research may be faster than current clinical methods available, the difference in infrastructure between research and clinical settings may prove the technology to be inefficient in practice, which could hinder the transition of diagnostic methods into clinical laboratories. Therefore, it is necessary to consider what current infrastructure is available on site and what may be logical and practical investments to facilitate the implementation of new diagnostics.

Despite the perceived barriers faced in hospitals delaying metagenomic implementation, it is worth noting that participants were enthusiastic about the potential of new diagnostics in laboratories to be integrated into the suite of tools already offered for infectious diseases. While COVID-19 was challenging for researchers and clinicians, the pandemic positively reinforced the benefits of utilising metagenomics to identify and monitor infectious diseases. High-profile outbreaks and infectious disease events result in an influx of funding and expedite the turnaround time for receiving accreditation, which allows for fast implementation of new technology in clinical settings. Applying metagenomics to clinical samples enables the identification of rare or unknown agents of infectious disease [38, 39]. In 2022, the UK government implemented a plan for integrating genomics into mainstream healthcare. It followed the 2020 Genome UK: The Future of Healthcare, which outlined an approach to becoming a world leader in genomic healthcare [26]. This was further supported by the most recent UKHSA publication, Pathogen Genomics Strategy, which emphasised the importance of genomics and its utility in healthcare, setting out guidance for its implementation, which included metagenomics for surveillance and diagnostic purposes [29] By bringing genomics to the forefront of diagnostics in healthcare, there is the opportunity to build capacity for the routine use of metagenomics in infectious disease, which will improve guidance for patient treatment and facilitate disease surveillance that is not already part of standard surveillance programmes [26].

mNGS has shown great potential for the detection of pathogens of infectious diseases, providing a sensitive method that can profile taxonomy, antimicrobial resistance and virulence through several clinical pilot studies and case reports [40–42]. However, evidence of the cost-effectiveness is also required to provide an argument for its added value to clinical laboratories. The operational value must be presented to inform clinical management of the benefit of technologies such as mNGS [14]. Evidence must be provided to highlight the method’s performance compared to currently available diagnostics and its impact on clinical decision-making. The cost-effectiveness of mNGS could be argued through a cost-benefit analysis, which would consider the economic benefit of mNGS and its public health impact through Quality Adjusted Life Years and an overall number of infections, similar to that published by Elliot et al. [43].

### Implications for policy and practice

The study’s findings demonstrated the transformative potential of metagenomics sequencing as an IDD, with participants enthusiastic about the future of the diagnostic landscape as genomic diagnostic methodologies are refined to suit the clinical needs of detecting infectious disease pathogens. First and foremost, for metagenomics to be successfully integrated into routine clinical use, a sustained funding stream to facilitate access to metagenomics and other novel diagnostic methods is equitable across sites and could reduce key implementation barriers. In addition, investment in the diagnostic infrastructure, including suitable training programs to upskill the current workforce or provide staff with the relevant expertise to deliver a genomics-focused diagnostic workflow, would support implementation. Workflows such as Oxford Nanopores EPI2ME pipeline have been developed to streamline bioinformatic analysis of clinical samples, including microorganism characterisation and antimicrobial resistance genes [8]. These workflows alleviate some of the bioinformatic burden faced by clinical laboratories by simplifying data processing. However, a baseline level of bioinformatics knowledge is still required for result interpretation, quality control, and troubleshooting. Therefore, there is still the requirement for dedicated training, such as clinical bioinformatics training as part of the NHS Scientist Training Programme and up upskilling of clinical laboratory staff to facilitate the implementation of mNGS in routine diagnostics.

Furthermore, for metagenomics-based IDD to be valuable, there could be standardised and accredited workflows to ensure the validity and rigour of the protocol and reporting of data. These priorities align closely with Strategic Aim 1 of the Pathogen Genomic Strategy developed by UKHSA, emphasising metagenomic utility for surveillance of ongoing and future outbreaks and metagenomic diagnostic capabilities and value [29]. The pathogen genomic strategy echoed the perspectives of academics and clinicians within this study that using genomics and metagenomics in the COVID-19 pandemic underscored the value of integrating these diagnostic methods into public health systems to support and inform clinical decision-making. Whilst investment is needed to expand mNGS capacity in clinical laboratories within hospitals, partnerships between hospitals and universities established during the COVID-19 pandemic could be a pragmatic interim solution. Academic collaborators could provide access to sequencing infrastructure, including bioinformatic expertise, storage and sequencing platforms for clinical teams. Leveraging these partnerships could help mitigate inequities in access observed during the study, particularly for smaller hospitals, while dedicated funding is sourced and training is developed to build capacity for in-house mNGS.

From a policy and practice perspective, a robust ethical framework for handling metagenomic data, which encompasses collection, storage and use of material, would support the clinical teams. This should be governed at the local and national levels, addressing concerns about a patient’s right to privacy and the value of sharing genomic data publicly. This would ensure genomic data is used responsibly and transparently. This could include national and locally sourced policies that set clear standards of practice for informed consent, data security, and access to metagenomic data where there is a public health benefit such as contributing to outbreak surveillance, understanding emerging new pathogens, outbreak response and AMR monitoring. In addition to physical policy implementation, an awareness of best practices for ethics in genomics improves an individual’s ethical preparedness. Ethical preparedness consists of three key behaviours, as described by Samuel et al. (2022), which include capability (understanding how to be ethically prepared), opportunity (factors within healthcare research and clinical settings which influence the ability to be ethically prepared) and motivation to sustain ethical behaviours [44]. Ethical preparedness goes beyond frameworks and policies; it establishes behaviours within individuals or groups to promote sustainable, ethical decision-making [45].

From a data security perspective, using strategies such as Secure Data Environments (SDEs) used by NHS England [46]. SDEs have transitioned away from data sharing between individuals, hospitals, or trusts to a data access platform to ensure data is stored and accessed safely and ethically. This move to SDE mitigates security and privacy concerns identified when using traditional data-sharing methods. The SDEs follow strict legal frameworks such as the Data Ethics Framework and Data Protection Act 2018 [47, 48].

In practice, it is clear from the participant’s experiences that improvement could be made in communicating within and between hospitals while fostering partnerships with other trusts and industry stakeholders to accelerate diagnostic innovation and metagenomic implementation in clinical settings. These partnerships would facilitate knowledge exchange, enabling a deeper understanding of best practices and the clinical application of metagenomic diagnostics. This knowledge could then contribute to a more informed plan for metagenomic diagnostic integration, learning from the experiences of other clinical laboratories. This could be further supported with cross-hospital training programs to facilitate staff upskilling in clinical metagenomics, including interpreting and reporting genomic data. Sharing training resources across institutions would reduce costs, enhance infrastructure development, and promote a standardised approach to metagenomic laboratory workflows and data analysis. Standardisation is particularly important, as variability in workflows and analysis methods has been identified as a key barrier to the widespread implementation of metagenomics in this study and the broader literature.

### Strengths and limitations

This study is the first of its kind to explore the perspectives of individuals involved with clinical metagenomics as an IDD. One of the key strengths was the diversity of expertise across the researchers, spanning public health policy and genomics in both research and clinical settings, which ensured that the question guide was reviewed from various perspectives. Although participants self-identified their level of expertise, there would be no particular reason for them to under or overestimate their own expertise, and based on the interview data, it is clear a diversity of experience was represented. In addition, having diverse expertise across the research team helped inform interpretation and improve the overall data analysis of the study. Recruitment took place in various locations across England, covering six regions, and included several stakeholders, allowing us to explore the varying perspectives across diverse professions associated with IDD. However, the Southwest and Northeast of England did not have representation among the participants. While several drivers are key defining features found across all interviews without representation, it is unknown if the findings apply to England’s two regions without representation. In addition, the study did not engage with participants in Wales, Northern Ireland or Scotland. This may have been due to the time when interviews were conducted during COVID-19. To further improve this study, further recruitment could be done to extend the recruitment beyond the regions covered to other locations within England to increase the representation of the cohort. In addition, recruitment could be expanded beyond England to include other countries in the UK and further afield internationally. By doing so, current diagnostic landscapes could be compared across countries, facilitating the identification of unique and shared barriers for genomic and metagenomic implementation in clinical settings as a routine diagnostic, as well as positive experiences within clinical settings to recognise best practices, such as those identified by P9 within this investigation. Finally, with larger numbers of participants, it may have been interesting to compare how perspectives might differ between clinical and academic participants, but with small numbers and many holding dual roles, there were no clear differences in perspectives in our data.

## Conclusions

The study addresses a significant gap in the qualitative literature around what is known about the development and implementation of metagenome sequencing for routine clinical use, taking an interpretive phenomenological approach. Identifying barriers and facilitators to implementing new technologies, particularly focusing on metagenomics into routine clinical use for IDD. It provided valuable insights into the user requirements of new diagnostics to integrate these tools into the current diagnostic landscape. Participants’ experiences of existing diagnostic workflows and the bottlenecks within them highlighted key drivers that could be considered when developing new diagnostics fit for purpose, such as cost, speed, and infrastructure.

The perspectives of key stakeholders informed political and practical recommendations to support the integration of metagenomics into routine clinical practice to improve patient care and public health. These considerations included establishing ethical frameworks for data and sample handling, improving stakeholder communication and partnerships, and developing collaborative training programs to upskill staff. In conclusion, the study highlights the utility of qualitative research in healthcare, particularly as applied to integrating metagenomics, demonstrating its capacity to uncover the added value of this technology as a diagnostic tool. Drawing on such insights, it emphasises the potential of incorporating new genomic technologies into clinical settings to enhance patient care and public health outcomes.

## Electronic supplementary material

Below is the link to the electronic supplementary material.

Supplementary Material 1

Supplementary Material 2

## Data Availability

The dataset (which includes individual transcripts) is not publicly available due to confidentiality.

## Abbreviations

COREQ: Consolidated criteria for reporting qualitative research
COG- UK: COVID-19 Genomic Consortium UK
IDD: Infectious disease diagnostics
IPA: Interpretive phenomenological approach
MALDI-TOF: Matrix-assisted laser desorption-ionization time of flight mass spectrometry
mNGS: Metagenomic next-generation sequencing
NHS: National Health Service
PCR: Polymerase Chain Reaction
SDEs: Secure Data Environments
UKHSA: United Kingdom Health Security Agency
