# Supplementary material for: Academic and clinical perspectives of metagenome sequencing as a diagnostic tool for infectious disease: an interpretive phenomenological study
Source: BMC Infect Dis. 2025 Mar 31;25:448. doi: 10.1186/s12879-025-10820-x (PMC11959724; doi:10.1186/s12879-025-10820-x)
Supplement: Supplementary file 1 — Supplementary Material 1 [file 12879_2025_10820_MOESM1_ESM.pdf]

Clinical perspectives of metagenome sequencing as a diagnostic tool for infectious diseases.

Opening:

You have been selected as a participant in this study due to your knowledge and expertise in infectious diseases and diagnostic methods used in clinical settings today. Today's interview will be around one hour long, exploring your thoughts and opinions on current diagnostic methods and the potential deployment of metagenome sequencing as an alternative diagnostic tool for pathogen detection. Taking a semi-structured interview approach, the interviewer may ask further questions in addition to the questions outlined below to ensure rich data is obtained; because of this, the interview may take longer than 1 hour.

This data will be used to evaluate the current infrastructure implemented in a clinical setting and identify the key factors driving the implementation of new diagnostic methods. This will ensure that the metagenome sequencing framework produced matches the priorities and needs for diagnosis.

Questions:

1. Please introduce yourself and give some information about your professional background and experience.

2. Do you have any experience using metagenomic sequencing with a clinical sample to diagnose a patient? If so, could you expand on your experience?
    a. What was the motivation behind using metagenomic sequencing as opposed to traditional clinical methods for diagnosis?

3. What are your priorities when considering approaches to diagnosis?

4. Gold standard methods for infectious disease diagnostics have remained unchanged for many years. Why do you think there has been little change in using molecular diagnostic methods?

5. What do you think are the main reasons why metagenome sequencing has not been implemented as a standard practice in infectious disease diagnostics?

6. There are many key factors that must be taken into consideration when implementing a new approach to infectious disease diagnostics. Can you tell me which factors you believe to be the most important to consider?

7. Based on the factors you mentioned above, are there particular trade-offs you think are important to consider when producing a new diagnostic framework?

8. In the current conditions of the COVID-19 pandemic, there has been a push for the use of genomic sequencing to better understand the virus. What are your thoughts on its use in the pandemic?

    a. Do you think this will have consequences for using genomic sequencing of other pathogens?

9. In addition to using genomic sequencing for COVID-19, researchers have implemented metagenomic sequencing in low and middle-income countries. For example, during the Ebola outbreak in 2014, Oxford Nanopore Technologies were used to identify outbreak strains and explore relationships of previous outbreak lineages. What is your viewpoint on the successes of metagenome sequencing outside of the UK?

    a. Does this offer scope for applying similar methods in UK-based clinical settings in the future?

10. In your opinion, what would be the perceived advantages of introducing metagenomic sequencing for pathogen detection in clinical settings?

11. In your opinion, what would be the perceived limitations of metagenomic sequencing for pathogen detection in clinical settings?

12. Metagenome sequencing requires expertise in computational infrastructure and proficiency in data handling and analysis to understand the outputs from DNA sequencing. In your opinion, what training would be required to produce a

diagnostic team capable of working through the pipeline from sample handling to data analysis?

13. Do you think there is room in current diagnostic frameworks to implement a new framework for pathogen detection, and how well would healthcare professionals receive them?

14. Do you have any further comments you would like to add regarding the topic we discussed today?

15. Is there anything you think is important that I missed during the interview?
